# *TNFRSF1B* Gene Variants and Related Soluble TNFR2 Levels Impact Resilience in Alzheimer's Disease

**DOI:** 10.3389/fnagi.2021.638922

**Published:** 2021-02-25

**Authors:** Jagan A. Pillai, Gurkan Bebek, Maria Khrestian, James Bena, Cornelia C. Bergmann, William S. Bush, James B. Leverenz, Lynn M. Bekris

**Affiliations:** ^1^Department of Neurology, Cleveland Clinic, Cleveland, OH, United States; ^2^Lou Ruvo Center for Brain Health, Cleveland Clinic, Cleveland, OH, United States; ^3^Neurological Institute, Cleveland Clinic, Cleveland, OH, United States; ^4^Center for Proteomics and Bioinformatics, Case Western Reserve University, Cleveland, OH, United States; ^5^Department of Nutrition, Case Western Reserve University, Cleveland, OH, United States; ^6^Genomic Medicine Institute, Cleveland Clinic, Cleveland, OH, United States; ^7^Department of Neuroscience, Lerner Research Institute, Cleveland Clinic, Cleveland, OH, United States; ^8^Department of Quantitative Health Science, Lerner Research Institute, Cleveland Clinic, Cleveland, OH, United States; ^9^Department of Population and Quantitative Health Sciences, Case Western Reserve University, Cleveland, OH, United States

**Keywords:** Alzheimer disease, neuroinflammation, TNFRSF1B, resilience, executive function, MCI, digit span

## Abstract

Tumor necrosis factor receptor 2 (TNFR2) promotes neuronal survival downstream. This longitudinal study evaluated whether the *TNFRSF1B* gene encoding TNFR2 and levels of its soluble form (sTNFR2) affect Alzheimer disease (AD) biomarkers and clinical outcomes. Data analyzed included 188 patients in the Alzheimer's Disease Neuroimaging Initiative (ADNI) who had mild cognitive impairment (MCI) and AD dementia. Further, a replication study was performed in 48 patients with MCI with positive AD biomarkers who were treated at a memory clinic. Cerebrospinal fluid (CSF) sTNFR2 levels along with two related *TNFRSF1B* gene single nucleotide polymorphisms (SNPs) rs976881 and rs1061622 were assessed. General linear models were used to evaluate the effect of CSF sTNFR2 levels and each SNP in relationship to CSF t-tau and p-tau, cognitive domains, MRI brain measures, and longitudinal cognitive changes after adjustments were made for covariates such as *APOE* ε*4* status. In the ADNI cohort, a significant interaction between rs976881 and CSF sTNFR2 modulates CSF t-tau and p-tau levels; hippocampal and whole brain volumes; and Digit Span Forwards subtest scores. In the replication cohort, a significant interaction between rs976881 and CSF sTNFR2 modulates CSF p-tau. A significant interaction between rs976881 and CSF sTNFR2 also impacts Clinical Dementia Rating Sum of Boxes scores over 12 months in the ADNI cohort. The interaction between *TNFRSF1B* variant rs976881 and CSF sTNFR2 levels was noted to modulate multiple AD-associated severity markers and cognitive domains. This interaction impacts resilience-related clinical outcomes in AD and lends support to sTNFR2 as a promising candidate for therapeutic targeting to improve clinical outcomes of interest.

## Introduction

Alzheimer disease (AD) is paradigmatically characterized by the presence of amyloid β plaques and tau neurofibrillary tangles in the brain. However, the cognitive and behavioral phenotypes of AD and their related CSF and MRI biomarker signatures can vary among patients, and molecular factors beyond amyloid β and tau likely play a role in this heterogeneity (van der Vlies et al., 2009; Whitwell et al., 2012; Dubois et al., 2014; Risacher et al., 2017; Pillai et al., 2019a). Multiple studies have evaluated *APOE* ε4 and other genes that may be related to the various MRI and clinical endophenotypes of AD (Hohman et al., 2014; Saykin et al., 2015; Louwersheimer et al., 2016; Therriault et al., 2020). Elucidating the interplay between genetic factors and these clinical phenotypes is crucial to developing precision medicine interventions and understanding cognitive “resilience,” a term used to describe better-than-expected cognitive performance in relation to the degree of AD pathology (Negash et al., 2013; Arenaza-Urquijo and Vemuri, 2018).

Accumulating evidence supports that inflammation-related changes may play a role in AD (Perry et al., 2010; Wyss-Coray and Rogers, 2012). We recently reported that key inflammatory analytes in the CSF related to rapid cognitive decline are pro-inflammatory among patients with the mild cognitive impairment (MCI) stage of AD (MCI-AD) (Pillai et al., 2020), whereas a cell-protective analyte profile is noted in the CSF correlating to neurodegeneration markers (Pillai et al., 2019b). Among the analytes that showed consistently high correlation with CSF t-tau, p-tau, and neuron-specific enolase was CSF soluble tumor necrosis factor receptor 2 (sTNFR2) (Pillai et al., 2019b). TNFR2 activation has been shown to promote downstream antiapoptotic responses and protect against oxidative stress-induced neuronal death and neurodegeneration (Fischer et al., 2011; Dong et al., 2016). sTNFR2 results from alternative splicing/shedding from membrane bound TNFR2 and, upregulation of the TNFR2 receptor is thought to be reflected in elevated sTNFR2 levels during inflammation (DeBerge et al., 2015). The *TNFRSF1B* gene (also known as *TNFR2*) and its variants have previously been linked with inflammatory responses, inflammatory disease outcomes, and rate of cancer progression (Fairfax et al., 2011; Steenholdt et al., 2012; Singhal et al., 2016), but the role of these gene variants in AD has yet to be elucidated.

We therefore sought to determine whether sTNFR2 and related *TNFRSF1B* gene variants affect the relationship between the biomarkers of tau pathology, MRI measures of disease severity, and cognitive outcomes. Our main hypothesis was that key *TNFRSF1B* gene variants and associated CSF sTNFR2 levels would modify AD biomarker levels of t-tau and p-tau, MRI brain volumes, and cognitive outcomes. After performing this analysis for patients enrolled in the Alzheimer's Disease Neuroimaging Initiative (ADNI), we evaluated the reliability of the findings by assessing the same factors in a group of patients with MCI-AD who were treated at a memory clinic.

## Materials and Methods

For the ADNI study, all patient consent was obtained according to the Declaration of Helsinki, and the study was approved by local Institutional Review Boards. For the replication memory clinic cohort, all patients provided written informed consent, and CSF, plasma, and DNA samples were collected for the Lou Ruvo Center for Brain Health Aging and Neurodegeneration Biobank following approval by the Cleveland Clinic Institutional Review Board.

### Study Design

A multistage study design was used to collect and replicate findings in two independent cohorts (the ADNI cohort and the replication memory clinic cohort) for which data regarding *TNFRSF1B* variants, CSF sTNFR2, and CSF AD biomarkers and cognitive variables were available. The CSF data used in this analysis were cleaned and quality controlled according to methodology described previously^1^.

### ADNI Cohort

ADNI is a longitudinal multicenter study designed to develop clinical, imaging, genetic, and biochemical biomarkers for the early detection and tracking of AD. The eligibility criteria for ADNI-1 (the first phase of the project) are described in the ADNI-1 protocol (available at http://adni.loni.usc.edu/methods/documents/). Briefly, eligible participants were aged 55 to 90 years, spoke either English or Spanish, and had an informant who was able to provide an independent evaluation of functioning. Eligible participants had also completed at least 6 years of education (or had a work history sufficient to exclude intellectual disability).

Our final analysis cohort consisted of 188 patients from ADNI-1 for whom CSF sTNFR2 levels and genetic status related to the *TNFRSF1B* SNPs of interest were available. In addition CSF Aβ42 levels were available for 177 and t-tau and p-tau levels for 182 of these patients. Details regarding the Elecsys method used to measure ADNI AD biomarkers are provided elsewhere (Shaw et al., 2019).

### ADNI Cohort: sTNFR2 Levels

Levels of sTNFR2 were measured in ADNI CSF samples using the RBM Discovery Multi-Analyte Profiling (MAP) v.1.0 panel (Myriad Genetics, Salt Lake City, Utah), which uses a Luminex platform (Myriad Genetics). Data obtained from “Biomarkers Consortium CSF Proteomics Project RBM Multiplex Data and Primer.”

### ADNI Cohort: Genotyping

ADNI-1 patients were genotyped using Illumina's10 Human610-Quad BeadChip (San Diego, California), and intensity data were processed with GenomeStudio v2009.1 (Illumina). For this study, we analyzed two *TNFRSF1B* SNPs: rs976881 and rs1061622, which were found to be in linkage equilibrium in all populations (*R*^2^ = 0.022 and D′ = 0.50; chi square = 108.34; *p* < 0.0001) according to LDlink analysis (Machiela and Chanock, 2015). These two SNPs are located within or in close proximity to the *TNFRSF1B* gene in the chr1p36 intronic 5' region (rs976881) and a missense coding variant in exon 6 (rs1061622). These SNPs were chosen based on their association with *TNFRSF1B-*related clinical outcomes and peripheral sTNFR2 levels (Glossop et al., 2005; Vistoropsky et al., 2010; Medrano et al., 2014; Cohen-Woods et al., 2018). Reference, heterozygous, and alternate alleles were T/T, T/C, and C/C, respectively, for rs976881, and G/G, G/T, and T/T, respectively, for rs 1061622. The reference and alternate allele notations were determined per the ADNI dataset based on calls from the International HapMap Project phase 3 data (Biffi et al., 2010). *APOE* ε*4* genotyping data were obtained from the ADNIMERGE table.

### ADNI Cohort: Structural MRI Acquisition and Processing

In ADNI-1 patients with MCI or dementia, results from 1.5T MRI scans were performed at baseline, and these results (taken from the ADNIMERGE primary table) were used for this analysis (Jack et al., 2008). The whole brain, ventricular volume, and hippocampal volume were the primary dependent variables, with intracranial volume used as a covariate in statistical models. These data are available as part of the ADNIMERGE package (downloaded on May 6, 2020).

### ADNI Cohort: Neurocognitive Measures

Baseline neurocognitive scores for ADNI-1 patients with MCI or dementia were analyzed. All major cognitive domains included in ADNI were evaluated, including the Logical Memory delayed recall score to assess verbal episodic memory (Wechsler Memory Scale, Logical Memory subtest) (Wechsler, 1981). Attention was assessed using the Digit Span subtest (Digit Span Forward and Digit Span Backward), and executive functioning was quantified using the Trail Making Test Part B, both from the Wechsler Adult Intelligence Scale (Wechsler, 1981). Object naming was assessed using the Boston Naming Test (Kaplan et al., 1983), and verbal fluency was assessed using a category fluency test (animals) (Strauss et al., 2006). The Mini Mental State Examination (MMSE) (Folstein et al., 1975) and Clinical Dementia Rating Sum of Boxes (CDR-SB) score (Morris, 1993) were used to assess global cognition, and change in CDR-SB score over 12 months was used to assess longitudinal change in cognition.

### Replication Memory Clinic Cohort

A cross-sectional replication cohort consisting of 48 patients in the MCI stage of AD (MCI-AD) were recruited from a specialized memory clinic at Cleveland Clinic (Lou Ruvo Center for Brain Health, Cleveland site). Recruitment details have been described previously (Pillai et al., 2019b).

The diagnosis of MCI-AD in these patients was confirmed by the presence of CSF Aβ42 and p-tau levels consistent with a diagnosis of AD as the primary etiology; the diagnosis was also based on consensus evaluation from two neurologists using published criteria (Albert et al., 2011). A commercially available test (ADmark Alzheimer's Evaluation, Athena Diagnostics, Marlborough, Massachusetts) was used to measure CSF levels of Aβ42, t-tau, and p-tau. All patients met the CSF cutoffs of ≤ 530 pg/mL for Aβ42 and ≥60 pg/mL for p-tau, which are consistent with amyloid-positive status on Amyvid (florbetapir F18 injection) at our center. *APOE* status was determined with blood samples (10 ng per patient) dispensed into 96-well plates for TaqMan (Applied Biosystems, Waltham, Massachusetts) allelic discrimination assays for the detection of SNPs associated with *APOE* alleles (*rs429358, rs7412*). PCR was performed using a 9700 Gene Amp PCR system (Applied Biosystems) and an end-point read in a 7500 Real-Time PCR system (Applied Biosystems) (Bekris et al., 2008).

### Replication Memory Clinic Cohort: sTNFR2 Levels

CSF levels of sTNFR2 were assessed as described previously (Pillai et al., 2019b). In brief, CSF was collected and analyzed by an independent laboratory via the RBM HumanMAP v.2.0, which is a subset of the RBM DiscoveryMAP v.1.0 used in the ADNI cohort; these platforms have the same quality control and thresholding process and are comparable. The lowest detectable dose of sTNFR2 was 0.0017 ng/mL. Samples of CSF were frozen within 15 min after collection and were processed (at −70°C in dry ice) and maintained at −80°C (in a non-frost-free refrigerator). The samples were shipped frozen in a Styrofoam container with sufficient dry ice to maintain the temperature at < -70°C for at least 48 h. Samples therefore underwent a single freeze-thaw cycle before analysis.

### Replication Memory Clinic Cohort: Genotyping

Genomic DNA was extracted from peripheral whole blood using standard protocols. Two *TNFRSF1B* SNPs (rs976881 and rs1061622) were characterized. *TNFRSF1B* variant allelic discrimination was performed using the 7500 Real Time PCR System and TaqMan SNP Genotyping Assays. The genotypes were determined based on sample clustering using the autocaller function of the Genotyping Application within Thermo Fisher Connect software.

### Replication Memory Clinic Cohort: Cognitive and Functional Measures

MMSE and Montreal Cognitive Assessment (MoCA) scores (Nasreddine et al., 2005) obtained <6 months before AD biomarker testing were available for all patients. In addition baseline CDR-SB scores (Morris, 1993) were available to characterize patients' cognitive and functional deficits.

### Statistical Analysis

Participants in ADNI-1 who had either MCI or AD dementia at baseline were merged into one group to ensure adequate power of analysis. The normality of biomarkers was evaluated using graphical methods and the Shapiro-Wilk test. A log (base 2) transformation allowed Pearson correlations to be fit; the sTNFR2 and AD biomarker levels described are therefore dimensionless. Along with estimates of correlation, 95% CIs and *p*-values were calculated. ANOVA models were used to estimate the mean differences between CSF sTNFR2 and t-tau and p-tau levels among reference, alternate, and heterozygous alleles of the *TNFRSF1B* SNPs. Multivariate general linear models were used to test the effect of CSF sTNFR2 levels and each of the two *TNFRSF1B* SNPs on CSF t-tau and p-tau, MRI brain measures, and cognitive domains after adjustments were made for covariates such as age, sex, years of education, *APOE* ε*4* status, and CSF Aβ42/p-tau ratio. Baseline MMSE and t-tau were also included as covariates in evaluations of cognitive domains as dependent variables, and baseline intracranial volumes and t-tau were included as covariates in evaluations of MRI measures as dependent variables.

First, we tested the effect of sTNFR2 levels on CSF t-tau and p-tau, MRI volumetric measures, and cognitive variables. Second, we tested the effect of each *TNFRSF1B* variant of interest on CSF t-tau and p-tau, MRI volumetric measures, and cognitive variables. Third, we tested whether the interaction effects between sTNFR2 and each *TNFRSF1B* variant and between sTNFR2 and t-tau were present for CSF t-tau and p-tau, MRI volumetric measures, and cognitive variables. Levene's test of equality of error variances, lack-of-fit F-test, and residual plots were used to assess model linearity and fit.

**Model 1**, evaluated the dependent variables CSF t-tau and p-tau. The main effects were CSF sTNFR2, *TNFRSF1B* SNP, and interaction effect between sTNFR2 and *TNFRSF1B* SNP. Covariates for this model included age, sex, CSF Aβ42, and *APOE*ε*4* status.

**Model 2**, evaluated the dependent variables hippocampal volume, ventricular volume, and whole brain volume. The main effects were CSF sTNFR2, *TNFRSF1B* SNP, CSF t-tau, and interaction effects between sTNFR2 and *TNFRSF1B* SNP and between sTNFR2 and t-tau. Covariates for this model included age, sex, years of education, CSF Aβ42/p-tau ratio, *APOE* ε*4* status, and baseline intracranial volume.

**Model 3**, evaluated the dependent variables Logical Memory delayed recall score, Digit Span subtest score, Trail Making Test Part B score, category fluency test (animals), and Boston Naming Test score. The main effects were CSF sTNFR2, *TNFRSF1B* SNP, CSF t-tau, and interaction effects between sTNFR2 and *TNFRSF1B* SNP and between sTNFR2 and t-tau. The covariates for this model included age, sex, years of education, CSF Aβ42/p-tau ratio, *APOE* ε*4* status, and baseline MMSE score.

**Model 4**, CDR-SB scores were log transformed because Levene's test demonstrated that the variances were significantly different for CDR-SB scores for the *TNFRSF1B* SNPs. A univariate general linear model was used to evaluate the interaction effect for each *TNFRSF1B* SNP and CSF sTNFR2 on change in CDR-SB score over 1 year in the ADNI cohort. CDR-SB score at 1 year was the dependent variable; the main effects were disease state (MCI or AD dementia), CSF t-tau, and interaction effect between sTNFR2 and *TNFRSF1B* SNP. The covariates included age, sex, years of education, *APOE* ε*4* status, baseline CDR-SB score, and CSF Aβ42/p-tau.

### Replication Cohort Model

Paralleling Model 1, here the dependent variables were again CSF t-tau and p-tau. The main effects were CSF sTNFR2, *TNFRSF1B* SNP, and interaction effect between sTNFR2 and *TNFRSF1B* SNP. Covariates for this model included age, sex, CSF Aβ42, and *APOE* ε*4* status.

Sensitivity analysis with and without covariates were conducted. Proportion of variance associated with one or more main effects was calculated using eta squared. All tests were two-tailed, and statistical significance was set at *p* < 0.05. For model 3 (with more than three dependent variables in the outcome), the Benjamini-Hochberg false discovery rate was used to assess significance. IBM SPSS Statistics for Windows, Version 22.0 (Armonk, New York) and RStudio Team (2020) RStudio (Version 1.2.5042) were used for all analyses.

### Data Availability

ADNI data are available on request at loni.ADNI.org.

## Results

Our analysis included a total of 188 ADNI-1 participants (59 with AD dementia; 129 with MCI) (Table 1).There were no differences in sTNFR2 or in CSF Aβ42, t-tau, or p-tau biomarker levels within the allelic subgroups of rs976881 or rs1061622.

**Table 1:** Demographics of ADNI cohort with patients divided into allelic subgroups, Mean (Std dev), percent of total and counts are presented.

**ADNI cohort (MCI + AD dementia)**	**rs976881 Homozygous reference T/T *N* = 16**	**rs976881 Heterozygous T/C *N* = 78**	**rs976881 Homozygous alternate C/C *N* =94**	**rs976881 Total 188**	***P*-value**	**rs1061622 Homozygous reference G/G *N* = 8**	**rs1061622 Heterozygous G/T *N* = 45**	**rs1061622 Homozygous alternate T/T *N* = 130**	**rs1061622 Total 183**	***P*-value**
MCI, N/total	11/16	51/78	67/94	129/188	0.70	4/8	31/45	92/130	127/183	0.46
Age, yrs	71.01 (8.8)	73.53 (7.9)	76.50 (6.1)	188	0.014^*^	75.95 (9.7)	76.27 (5.9)	74.29 (7.5)	183	0.23
Female, %F	18.7%	35.9%	37.2%	66/188	0.35	37.5%	44.4%	31.5%	64/183	0.29
Education, yrs	15.31 (2.9)	15.64 (3.0)	15.86 (2.8)	188	0.75	14.88 (3.4)	16.16 (2.6)	15.62 (2.9)	183	0.66
APOE ε4 *+ve*	7	50	58	115/188	0.58	5	29	77	111/183	0.80
Log_2_ CSF Aβ42	9.42 (0.5)	9.22 (0.5)	9.32 (0.5)	171	0.48	9.50 (0.7)	9.28 (0.4)	9.28 (0.5)	166	0.87
Log_2_ CSF t-tau	8.17 (0.4)	8.34 (0.4)	8.25 (0.6)	182	0.36	8.56 (0.4)	8.36 (0.5)	8.24 (0.5)	177	0.24
Log_2_ CSF p-tau	4.76 (0.5)	4.99 (0.5)	4.89 (0.7)	188	0.39	5.18 (0.5)	5.01 (0.6)	4.88 (0.6)	177	0.36
Log_2_ CSF sTNFR2	−0.13 (0.1)	−0.11 (0.1)	−0.147 (0.1)	188	0.59	−0.136 (0.1)	−0.11 (0.1)	−0.13 (0.1)	183	0.86
MMSE	26.56 (2.7)	25.85 (2.2)	25.74 (2.5)	188	0.37	25.38 (3.6)	26 (2.4)	25.93 (2.3)	183	0.94
CDR-SB	2.18 (1.3)	2.59 (1.8)	2.36 (1.7)	188	0.53	3.31 (2.1)	2.53 (2.0)	2.32 (1.5)	183	0.22
CDR-SB change at 12 months	0.25 (0.9)	1.20 (1.8)	0.81 (1.2)	188	0.19	0.93 (1.8)	1.18 (1.8)	0.83 (1.3)	183	0.44
Logical memory delayed	3.13 (2.9)	2.58 (2.4)	2.79 (2.7)	188	0.70	3 (3.1)	2.56 (2.6)	2.75 (2.6)	183	0.62
Digit span forward length	6.31 (1.3)	6.55 (0.9)	6.33 (1.1)	188	0.35	6.38 (0.9)	6.49 (1.1)	6.38 (1.0)	183	0.82
Digit span backward length	4.13 (1.4)	4.46 (1.0)	4.39 (1.2)	188	0.46	4.62 (0.7)	4.87 (1.1)	4.24 (1.2)	183	0.007^*^
Trails B score	143.87 (87.7)	161 (90.4)	160.52 (84.9)	188	0.98	181.25 (106.6)	146.59 (78.6)	158.36 (86.8)	183	0.51
Category fluency Animals	15.69 (6.0)	14.13 (4.6)	14.65 (4.8)	188	0.62	15.13 (6.8)	15.49 (5.3)	14.27 (4.5)	183	0.39
Boston naming test Total	26.06 (4.1)	24.81 (4.5)	24.12 (5.6)	188	0.24	24.38 (3.2)	24.93 (6.0)	24.62 (4.6)	183	0.39

Independent samples Kurskal Wallis test p-values,

* *p < 0.05. All cognitive scores at baseline unless otherwise stated*.

A total of 48 participants with MCI-AD were included in the replication memory clinic cohort (Table 2). There were again no differences in sTNFR2 or in CSF t-tau, or p-tau biomarker levels within the allelic subgroups of rs976881 and rs1061622. The differences between ADNI and replication cohort is noted in Supplementary Table 1. Both cohorts had similar baseline CDR-SB scores, years of education and were predominantly White and a slightly higher frequency of men. The replication cohort had a lower age, higher frequency of *APOE* ε4 carriers and a slightly lower baseline MMSE.

**Table 2:** Demographics of replication memory clinic cohort with patients divided into allelic subgroups.

**Replication Cohort (MCI only)**	**rs976881 Homozygous reference T/T *N* = 8**	**rs976881 Heterozygous T/C *N* = 19**	**rs976881 Homozygous alternate C/C *N* = 21**	**rs976881 Total**	***P*-value**	**rs1061622 Homozygous reference G/G *N* = 2**	**rs1061622 Heterozygous G/T *N* = 38**	**rs1061622 Homozygous alternate T/T *N* = 8**	**rs1061622 Total**	***P*-value**
Age, yrs	67.9 (6.7)	69.6 (7.9)	64.3 (7.4)	48	0.088	65.0 (7.6)	66.8 (7.9)	67.3 (7.0)	48	0.65
Female, %F	22.2%	47.4%	45%	20/48	0.50	50%	39.5%	50%	20/48	0.90
Education, yrs	16.1 (2.4)	15.0 (3.1)	15.4 (2.9)	48	0.65	13.0 (1.4)	15.8 (2.7)	14.1 (3.3)	48	0.20
APOE ε4 *+ve*	5	16	15	36/48	0.46	2	28	6	36/48	0.72
Log _2_ CSF Aβ42	8.5 (0.26)	8.04 (0.47)	8.9 (0.63)	48	0.018^*^	8.0 (0.56)	8.2 (0.52)	7.7 (0.58)	48	0.12
Log _2_ CSF t-tau	8.7 (0.50)	9.3 (1.02)	8.7 (0.88)	48	0.066	9.07 (1.09)	8.9 (0.91)	9.7 (0.74)	48	0.41
Log _2_ CSF p-tau	6.2 (0.39)	6.6 (0.72)	6.1 (0.63)	48	0.075	6.8 (0.58)	6.3 (0.67)	6.4 (0.67)	48	0.41
Log_2_ CSF sTNFR2	1.06 (0.33)	1.21 (0.59)	1.25 (0.4)	48	0.54	0.96 (0.28)	1.19 (0.49)	1.2 (0.00)	48	0.32
MMSE Baseline	24.5 (3.7)	25.1 (3.1)	23.4 (6.1)	48	0.39	25.0 (1.4)	24.2 (5.1)	24.3 (3.3)	48	0.59
MOCA Baseline	19.3 (4.2)	18.2 (4.8)	18.2 (2.4)	48	0.79	13.0 (1.4)	18.5 (4.5)	18.0 (4.1)	48	0.14
CDR-SB Baseline	2.07 (0.87)	2.05 (1.30)	2.22 (1.22)	47	0.43	2.3 (1.8)	2.1 (1.2)	2.3 (1.1)	47	0.90

Mean (Std dev), percent of total and counts are presented. Independent samples Kurskal Wallis test p-values,

* *p < 0.05*.

### ADNI Cohort: Model 1

**Figure 1:**
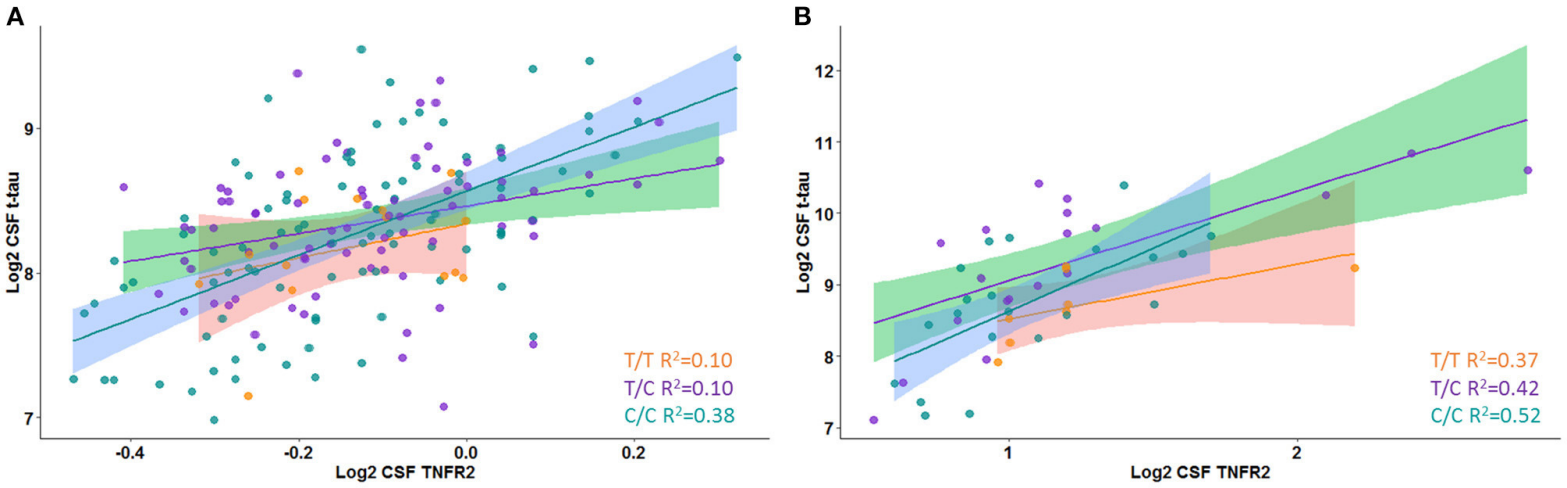
Interaction between rs976881 and soluble tumor necrosis factor receptor 2 (sTNFR2) and the effect on CSF t-tau in Alzheimer's Disease Neuroimaging Initiative (ADNI) and replication cohorts. Regression lines, 95% CI and *R*^2^ values shown for the alleles T/T, T/C, and C/C. **(A)** ADNI cohort. **(B)** Replication cohort.

A significant interaction effect between rs976881 and CSF sTNFR2 was associated with CSF t-tau levels, but this association with CSF t-tau levels was not seen for the interaction effect between rs1061622 and CSF sTNFR2 (Table 3 and Figure 1A). The effect sizes (by partial eta squared values) were 0.046 for CSF t-tau and 0.044 for CSF p-tau (i.e., 4.6% of all variance in CSF t-tau and 4.4% of all variance in CSF p-tau were attributable to the interaction effect between rs976881 and CSF sTNFR2). The T/T group was noted to have a shallower slope and poorer fit (*R*^2^ = 0.1) than C/C (*R*^2^ = 0.38), suggesting that sTNFR2 levels are not as closely related to t-tau levels in T/T as in C/C. The direct main effects of sTNFR2 on CSF t-tau and p-tau are shown in Supplementary Table 2.

**Table 3:** Key results of the general linear models: Results from the ADNI and replication memory clinic cohorts, *p ≤ 0.05.

Rs976881 * sTNFR2	**Df**	**Mean Square**	**F**	***P*-value**	**Adjusted *R*^**2**^**	**Partial Eta squared**
**Model 1:** Dependent variables: CSF t-tau and p-tau, Main effects: CSF sTNFR2, TNFRSF1B SNP, Interaction effect: sTNFR2 X TNFRSF1B SNP, Covariates: age, sex, CSF Aβ42, APOEε4 status						
**ADNI cohort**						
Log CSF t-tau	2,165	0.21	3.94	0.021*	0.27	0.046
Log CSF p-tau	2,165	0.33	3.83	0.024*	0.24	0.044
**Replication cohort**						
Log CSF t-tau	2,44	1.30	2.82	0.07	0.47	0.11
Log CSF p-tau	2,44	0.70	3.20	0.05*	0.51	0.13
Rs1061622*sTNFR2						
Log CSF t-tau	2,44	1.60	3.58	0.036*	0.48	0.14
Log CSF p-tau	2,44	0.77	3.56	0.037*	0.52	0.14
**Model 2:** Dependent variables: hippocampal volume, ventricular volume and whole brain volume, Main effects: CSF sTNFR2, *TNFRSF1B* SNP, CSF t-tau, Interaction effects: sTNFR2 X *TNFRSF1B* SNP and sTNFR2 X t-tau, Covariates: age, sex, education, CSF Aβ42/p-tau ratio, *APOEε4* status and baseline intra cranial volume						
**ADNI Cohort**						
Hippocampus	2,134	2646708.94	6.48	0.002*	0.08	0.088
Ventricles	2,134	33816326.80	0.64	0.94	0.12	0.001
Whole brain volume	2,134	9426117372.29	4.21	0.017*	0.16	0.059
**Model 3:** Dependent variables: Logical Memory delayed recall, Digit Span length, Trails B score and Boston Naming total score, Main effects:CSF sTNFR2, TNFRSF1B SNP, CSF t-tau, Interaction effects: sTNFR2 X TNFRSF1B SNP and sTNFR2 X t-tau, Covariates: age, sex, education, CSF Aβ42/p-tau ratio, APOEε4 status and baseline MMSE						
**ADNI Cohort**						
Logical memory	2,159	5.22	0.78	0.459	0.038	0.01
Digit span forward length	2,159	8.90	8.89	<0.0001*	0.064	0.099
Digit span backward length	2,159	4.43	4.43	0.036*	0.025	0.041
Trails B-score	2,159	26889.98	3.71	0.027*	0.046	0.045
Category Fluency Animals	2,159	92.64	3.96	0.021*	0.025	0.047
Boston naming test	2,159	22.61	0.84	0.43	0.003	0.01

### ADNI Cohort: Model 2

A significant interaction effect between rs976881 and CSF sTNFR2 was associated with hippocampal volume and whole brain volume but not with ventricular volume (Table 3). 8.8% of all variance in hippocampal volume and 5.9% of all variance in whole brain volume were attributable to the interaction effect between rs976881 and CSF sTNFR2.

The MRI measures were not affected by rs1061622, nor by the interaction effect between sTNFR2 and CSF t-tau. CSF t-tau did have a significant direct main effect on ventricular volume and whole brain volume but not on hippocampal volume (Supplementary Table 2).

### ADNI Cohort: Model 3

**Figure 2:**
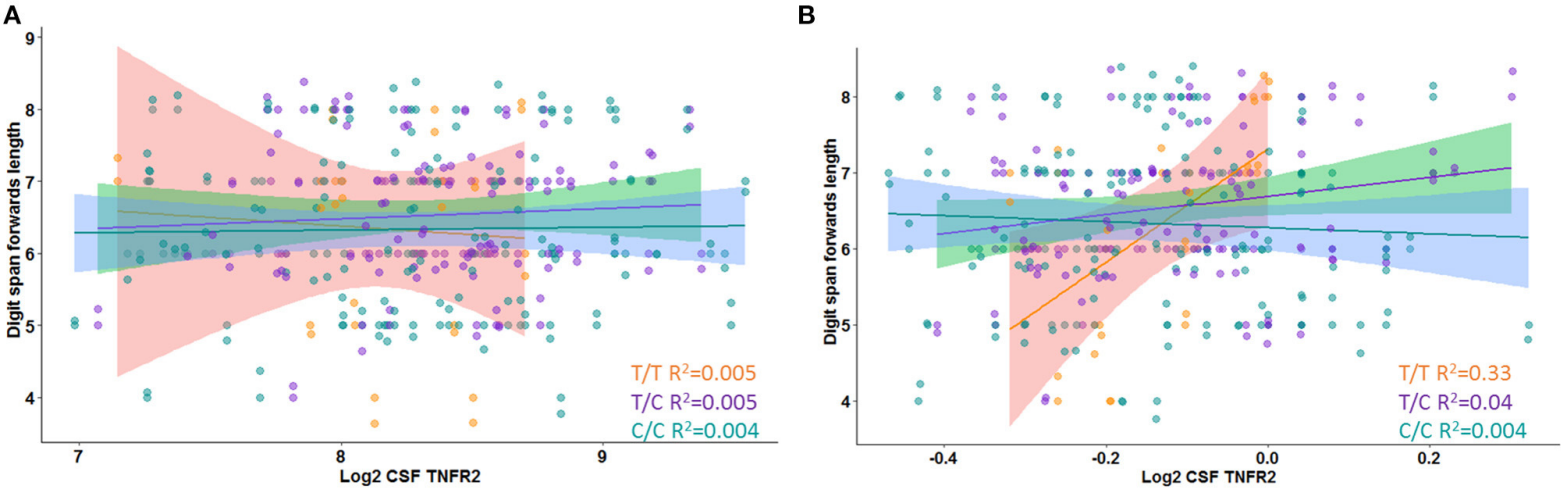
Interaction between rs976881 and soluble tumor necrosis factor receptor 2 (sTNFR2) and between rs976881 and CSF t-tau and the effect on Digit Span Forward score. **(A)** CSF t-tau **(B)** CSF sTNFR2. Regression lines, 95% CI and *R*^2^ values shown for the alleles T/T, T/C, and C/C. Alzheimer's Disease Neuroimaging Initiative (ADNI) data.

In Model 3, a significant interaction effect between rs976881 and CSF sTNFR2 was associated with the Digit Span subtest score, the Trail Making Test Part B score, and the category fluency test (animals) score (Table 3). 9.9% of all variance for the Digit Span Forward score and 4.7% of all variance for the category fluency (animals) score were attributable to the interaction effect between rs976881 and CSF sTNFR2. There was no significant interaction effect between sTNFR2 and CSF t-tau, nor was there a significant main effect of CSF t-tau on these cognitive measures (Figure 2A). As shown in Figure 2B, the T/T group had a steeper slope and a larger regression coefficient for the Digit Span Forward score (*R*^2^ = 0.33), suggesting that lower sTNFR2 levels (but not CSF t-tau levels) relate to poorer Digit Span Forward scores for this group, unlike for the C/C group (*R*^2^ = 0.004). The interaction effect between rs1061622 and CSF sTNFR2 had no effect on these cognitive measures. The Digit Span Forward score was significant even after false discovery rate correction (false discovery rate–corrected *p*-values are provided in the Supplementary Material).

### ADNI Cohort: Model 4

**Figure 3:**
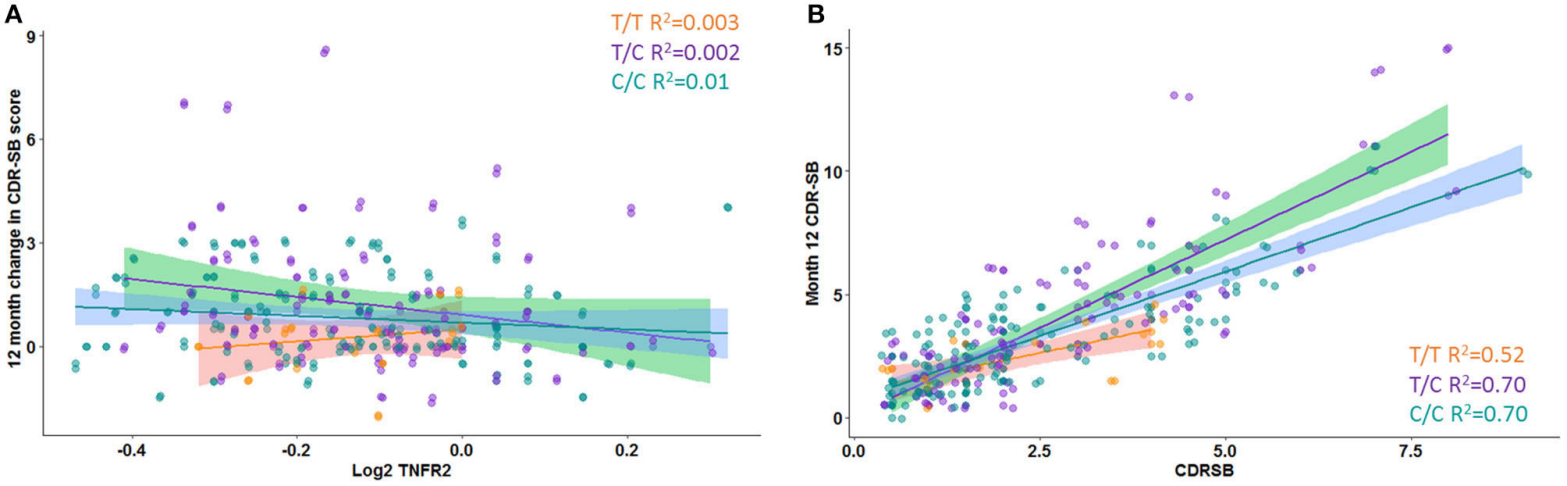
Change in Clinical Dementia Rating Sum of Boxes (CDR-SB) score over time. **(A)** Interaction between rs976881 and sTNFR2 level and the effect on change in CDR-SB score over 1 year. **(B)** Slope of change in score over 1 year. Regression lines, 95% CI and *R*^2^ values shown for the alleles T/T, T/C, and C/C. Alzheimer's Disease Neuroimaging Initiative (ADNI) data.

A significant interaction effect between rs976881 and sTNFR2 was associated with change in CDR-SB scores over 1 year (df 3, 175; mean square = 0.21; *F* = 2.83; adjusted *R*^2^ = 0.36). A total of 4.6% of all variance in CDR-SB change over 1 year was attributable to the interaction effect between rs976881 and CSF sTNFR2 (Figure 3A). As shown in Figure 3B, the T/T group had a smaller change (shallower slope) in CDR-SB change over 1 year than the C/C group (R^2^ = 0.52 vs 0.70). There was no significant effect of rs1061622 on CDR-SB change over 1 year (df 3, 170; *F* = 2.32; adjusted *R*^2^ = 0.35).

### Replication Memory Clinic Cohort

A significant interaction effect between rs976881 and CSF sTNFR2 was associated with CSF p-tau levels (Table 3). Eleven percentage of all variance in CSF t-tau and 13% of all variance in CSF p-tau were attributable to the interaction effect between rs976881 and CSF sTNFR2. The homozygous reference (T/T) group had a shallower slope and a smaller regression coefficient (*R*^2^ = 0.37) than the homozygous alternate allele (C/C) (*R*^2^ = 0.42) (Figure 1B), suggesting that sTNFR2 levels are not as closely related to t-tau levels in the T/T replication memory clinic group as they are in the ADNI cohort. Pairwise comparisons for the rs976881 alleles demonstrated significant differences between the T/T and T/C groups in CSF t-tau levels (mean difference = −0.58; 95% CI: 0.074 to 1.09) and CSF p-tau levels (mean difference = −0.39; 95% CI: 0.050–0.747). Supplementary Table 2 provides details regarding the direct main effects of significance.

A significant interaction effect between rs1061622 and CSF sTNFR2 was associated with CSF t-tau and p-tau levels. Fourteen percentage of all variance in CSF t-tau and 14% of all variance in CSF p-tau were attributable to the interaction effect between rs1061622 and CSF sTNFR2 (Table 3). Pairwise comparisons for the rs1061622 alleles demonstrated significant differences between the T/T and T/G groups in CSF t-tau levels (mean difference = −0.69; 95% CI: −0.079 to −1.29) and CSF p-tau levels (mean difference = −0.49; 95% CI: −0.067 to −0.91).

## Discussion

Immune-related genes and pathways have been found to play a role in AD pathophysiology (Fischer et al., 2011; Kunkle et al., 2019). Previous studies have assessed how these genetic immune and factors could affect AD biomarkers (Hohman et al., 2014; Louwersheimer et al., 2016; Deming et al., 2019) and clinical outcomes (Pillai et al., 2020), but little research has addressed their potential contribution to resilience. The inflammatory analyte TNF has been found to play a role in synaptic plasticity and modulating responses to neural injury and neurodegeneration (Beattie et al., 2002; Ellwardt et al., 2018), suggesting that it may also be involved in cognitive resilience (Pape et al., 2019). The current study demonstrated that the interaction between TNF pathway–related sTNFR2 CSF levels and *TNFRSF1B* gene variants contributed to significant differences in AD-associated severity markers such as CSF t-tau and p-tau, MRI measures of interest, and cognitive scores in the ADNI cohort. In a replication memory clinic cohort, the interaction between CSF sTNFR2 and rs976881 was also associated with differences in CSF p-tau biomarkers, with a significantly larger effect size than was seen in the ADNI cohort. In the ADNI cohort, rs976881 T/T allele (homozygous reference) carriers demonstrated slower rates of CDR-SB score changes in relation to corresponding sTNFR2 levels. Additionally, statistical interaction effects demonstrated that the strength of a linear relationship between sTNFR2 and (1) CSF t-tau and p-tau, (2) MRI whole brain and hippocampal volumes, and (3) Digit Forward test score varied for the rs976881 T/T allele compared to the C/C and T/C alleles. Studies in animal models have demonstrated that TNFR2 plays a protective role against neurodegeneration in the CNS (Marchetti et al., 2004; Fischer et al., 2011; Dong et al., 2016). Taken together, these findings support this genotypic variant is a potential marker of resilience in AD.

sTNFR2 is expressed primarily in immune and endothelial cells. TNFR2 receptor signaling is involved in pro-survival signaling pathways, which activate cellular inhibitors of apoptosis and the NF-κB pathway (Kanehisa et al., 2016). Subsequent downstream activation of PKB/Akt promotes cell survival and proliferation (Fischer et al., 2020). sTNFR2 has previously been found to be highly correlated with CSF t-tau, p-tau, and neuron-specific enolase in MCI-AD (Pillai et al., 2019b), and a similar profile with *TNFRSF1B* activation was noted in a parallel brain transcriptome analysis (Pillai et al., 2019b). It is possible, therefore, that sTNFR2 and *TNFRSF1B* SNPs play a modulating role in regard to clinical outcomes in AD, rather than serving as an AD risk gene that have been the focus of prior genome-wide association studies in AD. This is consistent with prior reports of gene variants related to resilience in AD which suggest that genetic architecture of resilience appears to be distinct from that of clinical AD (Dumitrescu et al., 2020).

Research has shown that rs976881 and rs1061622 are associated with sTNFR2 levels in the periphery, with the rs976881 reference allele (T/T) related to higher sTNFR2 levels than the alternate allele (C/C) (Vistoropsky et al., 2010; Cohen-Woods et al., 2018). In the replication memory clinic cohort in this study, patients with the rs976881 reference allele T/T had lower CSF t-tau and p-tau levels than patients with T/C (given their corresponding CSF sTNFR2 levels). Previous research has also found that rs976881 T/T is less responsive than T/C to anti-TNFα maintenance therapy (infliximab) in patients with Crohn's disease (Steenholdt et al., 2012). It is therefore likely that among minor allele (T/T) carriers with Crohn's disease, higher levels of TNFR2 counteract TNF-α, making infliximab less effective. In our study, the effect of rs976881 may also be tied to its effect on sTNFR2 levels, with the rs976881 minor allele (T/T) demonstrating a more robust sTNFR2 response than C/C and T/C alleles to CSF t-tau and p-tau levels. Studies have also demonstrated that the minor allele of rs1061622 is also related to sTNFR2 levels and response to anti-TNFα maintenance therapy in patients with Crohn's disease (Medrano et al., 2014). In our study, the significant interaction observed between rs1061622 and sTNFR2 and its effect on CSF t-tau and p-tau in the replication cohort but not in the ADNI cohort suggests differences in the nature of participants recruited, as discussed in further detail later.

In the ADNI cohort, a significant interaction between rs976881 and CSF sTNFR2 was strongest with regards to Digit Span Forwards subtest, but was also noted in Trail Making Part B test, and category fluency test (animals) scores. These measures assess overlapping but distinct cognitive skills, including working memory, complex attention, and speed of information processing, and can be categorized as contributing to domains of attention/executive functioning and processing speed. In this cohort, rs976881 status was also related to the change in CDR-SB score over 1 year. This is consistent with our previous finding that better initial performance on the Digit Span subtest and related working memory task is associated with a slower rate of functional decline on the CDR-SB test over time in patients with AD (Pillai et al., 2014). Additionally, higher levels of pro-cell survival and inflammation resolution chemokines have been found in the human temporal cortex and entorhinal cortex at autopsy among human brains resilient to AD pathology (Barroeta-Espar et al., 2019). These results are reflected in our study, which demonstrated that a significant interaction between rs976881 and CSF sTNFR2 modulated favorable outcomes in three measures related to AD severity: CSF biomarkers of neurodegeneration and tau (CSF p-tau and t-tau), MRI measures (hippocampal and whole brain volumes), and cognitive measures (Digit Span Forward score and CDR-SB score).

Strengths/limitations: The current study demonstrated directional replication by consistently noting a similar directional interaction effect of sTNFR2 and rs976881 on CSF p-tau and t-tau in both the ADNI and replication cohorts. Additionally, the effect of rs976881 on MRI and cognitive outcomes demonstrated in this study is consistent with the known relationship between these biomarkers (CSF p-tau, t-tau, hippocampal volume, and whole brain volumes) and longitudinal cognitive outcomes in the ADNI population. In the ADNI cohort for this study, 4.6% of the variance in CSF t-tau levels was explained by rs976881 and sTNFR2, whereas 11% of the variance in CSF t-tau was attributed to the same variables in the replication cohort. There were some differences between the cohorts, however. First, the positive correlation between the sTNFR2 and neurodegeneration biomarkers are consistent within each cohort but the replication cohort had 2.2 times higher mean levels of p-tau than ADNI (Pillai et al., 2019b). Mean sTNFR2 levels correlating with neurodegeneration biomarkers were also 2.4 times higher in the replication cohort than ADNI. This is likely due to different participant characteristics for the two cohorts; for instance, the replication cohort was a sample of memory clinic patients with a faster rate of disease progression than patients in the ADNI cohort (Pillai et al., 2020). Second, the replication cohort included participants at the MCI stage of AD, whereas the ADNI cohort included patients with MCI and AD dementia. As the replication cohort was not harmonized with the ADNI cohort for all of the neurocognitive variables and MRI volumetric measures, there could not be a close corroboration between the replication cohort and the ADNI cohort for the multiple variables of interest. These results are likely generalizable to MCI and AD dementia subjects with positive AD biomarkers but the fact that patients in both cohorts were predominantly Caucasian and had a higher level of education suggests the need for replication of these results among other racial and ethnic cohorts with a diverse education and socioeconomic backgrounds.

Within the ADNI cohort, MRI hippocampal volumes were noted to have a significant interaction effect between rs976881 and sTNFR2, while logical memory delayed recall scores did not meet significance. One possible reason for this is that the predominance of amnestic patients with MCI despite likely multiple etiologies of amnestic complaints in ADNI cohort, limits our ability to discriminate based on logical memory delayed recall scores alone given normative limits (Nettiksimmons et al., 2014). Additional corroboration of these results in a cohort with recruitment goals different from those of ADNI is therefore warranted. The lack of neuropathologic confirmation of diagnosis also limits our understanding regarding the potential role of mixed pathology on clinical outcomes.

The study is an evaluation of the association between CSF sTNFR level and *TNFRSF1B* SNPS on clinical outcomes, and does not address the mechanistic relationship between them on neurodegeneration. This study further does not provide a comprehensive survey of *TNFRSF1B* SNPs in AD, as we did not analyze other *TNFRSF1B* SNPs reported to be related to sTNFR2 levels (e.g., rs590368). These other SNPs were not included because of the smaller number of participants within ADNI who had these genetic variants and who also had measured levels of sTNFR2 available. Using multiple SNPs may allow researchers to define a stronger *TNFRSF1B* haplotype with regard to sTNFR2 levels, and some variables may become more or less salient considering additional SNP interactions. Independent replication using the same biomarkers used in ADNI would allow for further clarification. Type II errors have to be considered for this study, given the small number of patients within some of the allele groups; smaller effect sizes could therefore have been missed.

The rs976881 T/T reference genotype has been related to higher levels of sTNFR2 previously and activation of TNFR2 signaling has been posited as a promising strategy for AD therapy (Ortí-Casañ et al., 2019). We have now demonstrated that interaction between *TNFRSF1B* gene variant rs976881 and CSF sTNFR2 affects CSF and MRI biomarkers of neurodegeneration, cognitive profiles, and rate of functional decline over 1 year. These results provide important information regarding the molecular characterization of AD phenotypes and suggest that this genotypic variant could be used as a marker of resilience in AD. Independent confirmation of these results in other cohorts with mutidomain AD biomarkers is warranted.

## Data Availability Statement

The datasets presented in this study can be found in online repositories. The names of the repository/repositories and accession number(s) can be found at: http://adni.loni.usc.edu/, genetic-data.

## Ethics Statement

The studies involving human participants were reviewed and approved by Cleveland Clinic Institutional Review Board ADNI Individual Site Institutional Review Board. The ethics committee waived the requirement of written informed consent for participation.

## Author Contributions

JP: obtained funding for research project, organization of research project, execution of research project, design the statistical analysis, execution of statistical analysis, and writing the first draft of manuscript preparation. GB: execution of research project, review and critique of statistical analysis, and review and critique of manuscript preparation. MK: execution of research project and review and critique of manuscript preparation. JB: design the statistical analysis and review and critique of manuscript preparation. CB, WB, and JL: review and critique of statistical analysis and review and critique of manuscript preparation. LB: organization of research project, review and critique of statistical analysis, and review and critique of manuscript preparation. All authors contributed to the article and approved the submitted version.

## Conflict of Interest

JP receives research funding from the National Institutes of Health, Alzheimer's Association, and Keep Memory Alive Foundation. CB receives research funding from the National Institutes of Health. WB receives research funding from the National Institutes of Health. JL has received consulting fees from Acadia, Aptnyx, Biogen, Eisai, GE Healthcare, Sanofi, and Takeda and grant support from the Alzheimer's Association, Alzheimer's Drug Discovery Foundation, Biogen, Department of Defense, GE Healthcare, Genzyme/Sanofi, Lewy Body Dementia Association, Michael J Fox Foundation, and National Institute of Health (NIA, NINDS). The remaining authors declare that the research was conducted in the absence of any commercial or financial relationships that could be construed as a potential conflict of interest.

## Footnotes

^1^*Biomarkers Consortium ADNI CSF Targeted Proteomics Project - Data Primer Version 28 Dec 2011*. Available online at: http://adni.loni.usc.edu/methods/ (accessed December 1, 2018).
