# Supplementary material for: TNFRSF1B Gene Variants and Related Soluble TNFR2 Levels Impact Resilience in Alzheimer's Disease
Source: Front Aging Neurosci. 2021 Feb 25;13:638922. doi: 10.3389/fnagi.2021.638922 (PMC7947258; doi:10.3389/fnagi.2021.638922)
Supplement: Supplementary file 1 [file Table_1.DOCX]

**Supplementary Table 1: Comparison of ADNI and replication cohorts**

|  | | |  |  |
| --- | --- | --- | --- | --- |
|  | **ADNI**  **N=188** | **Replication**  **N=48** | | **P value** |
| **MCI %** | 68.6% | 100% | | **<0.0001_c_** |
| **Age, yrs** | 74.8(7.3) | 68.1(7.3) | | **0.0001_a_** |
| **Education yrs** | 15.7[14.0,18.0] | 16.0[12.5,18.0] | | 0.27_b_ |
| **Female %** | 35.1% | 41.7% | | 0.31_c_ |
| **Race, % White** | 96.8% | 95.8% | | 0.7_c_ |
| ***APOE ε4 %*** | 61.2% | 77.1% | | **0.014_c_** |
| **Baseline CDR-SB** | 2.4(1.8) | 2.2(1.3) | | 0.47_a_ |
| **Baseline MMSE** | 25.9 (2.4) | 24.8(3.1) | | **0.012_a_** |
|  |  |  | |  |
| Statistics presented as Mean ± SD, Median [P25, P75] or *N* (column %).   - *P*‐values: a = t-test for continuous variables, b = Kruskal–Wallis test, c = Pearson's chi‐square test. In bold p<0.05   MMSE, Mini‐mental state exam; CDR‐SB, Clinical dementia rating scale‐sum of boxes | | | | |

**Supplementary Table 2:** Significant main effects results of the general linear models: Results from the ADNI and replication memory clinic cohort.


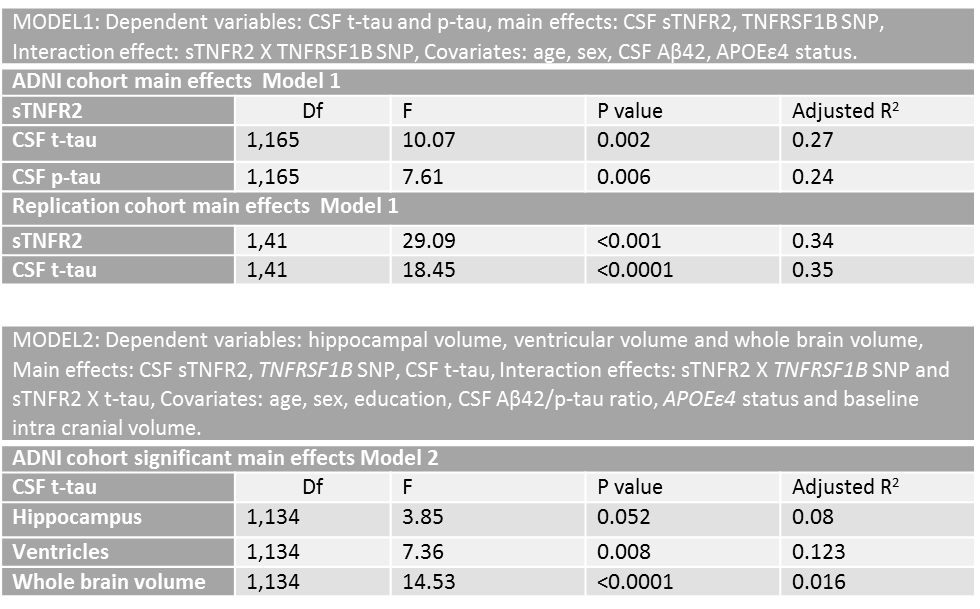


**Supplementary Material:**

The Benjamini-Hochberg adjusted P values for Model 3 are as follows:

Digit span forwards: 0.0006

Category fluency animals:0.063

Trails B score:0.054

Digit span backwards:0.054

Boston naming test:0.51

Logical memory:0.46
